# DCision-making in tumors governs T cell anti-tumor immunity

**DOI:** 10.1038/s41388-021-01946-8

**Published:** 2021-07-21

**Authors:** Francesca Alfei, Ping-Chih Ho, Wan-Lin Lo

**Affiliations:** 1 Department of Oncology, University of Lausanne, Lausanne, Switzerland; 2 Ludwig Institute of Cancer Research, University of Lausanne, Lausanne, Switzerland; 3 Division of Rheumatology, Rosalind Russell and Ephraim P. Engleman Arthritis Research Center, Department of Medicine, University of California, San Francisco, San Francisco, CA USA; 4 Division of Microbiology and Immunology, Department of Pathology, University of Utah, Salt Lake City, UT USA

**Keywords:** Innate immune cells, Tumour immunology

## Abstract

The exploitation of T cell-based immunotherapies and immune checkpoint blockade for cancer treatment has dramatically shifted oncological treatment paradigms and broadened the horizons of cancer immunology. Dendritic cells have emerged as the critical tailors of T cell immune responses, which initiate and coordinate anti-tumor immunity. Importantly, genetic alterations in cancer cells, cytokines and chemokines produced by cancer and stromal cells, and the process of tumor microenvironmental regulation can compromise dendritic cell–T cell cross-talk, thereby disrupting anti-tumor T cell responses. This review summarizes how T cell activation is controlled by dendritic cells and how the tumor microenvironment alters dendritic cell properties in the context of the anti-tumor immune cycle. Furthermore, we will highlight therapeutic options for tailoring dendritic cell-mediated decision-making in T cells for cancer treatment.

## Introduction

T cells mount remarkably sensitive and selective adaptive immune responses against pathogen-infected cells and neoplastic cells by recognizing short peptides presented by major histocompatibility complex (MHC) molecules. The degree to which a T cell response is elicited by only a specific peptide–MHC (pMHC) stimulus is referred to as T cell specificity. T cell specificity relies on proper self- versus non-self ligand discrimination by the T cell receptor (TCR). A tumor-specific T cell response requires TCR recognition of a tumor-specific or -associated antigen, or a cancer germline/cancer testis antigen [1–4] (and reviewed in detail in [5]). Mutant tumor neoantigens have attracted the greatest interest due to their potential in cancer immunotherapies.

Neoantigens are antigens derived from mutated self-proteins expressed by tumor cells that can be distinguished from the self-peptides of normal proteins in healthy tissues; thus, neoantigens are tumor-specific antigens. As most self-reactive T cells are deleted in the thymus via negative selection, mutant tumor neoantigens are potentially more immunogenic than typical self-antigens. The presentation of neoantigens supports tumor-specific T cell responses, and the tumor neoantigenome may influence anti-tumor T cell responses. Given that CD8 T cells are the main effector cells in anti-tumor responses, many studies have utilized next-generation sequencing and epitope prediction algorithms to identify and predict MHC class I (MHC-I)-restricted neoantigenomes. The MHC-I antigen presentation pathway processes mutant tumor proteins, which are loaded onto MHC-I molecules and presented to CD8 T cells, thereby enabling specific cytotoxic responses that eliminate tumor cells with the neoantigens on their cell surfaces. Consistent with this notion, some studies have shown that tumor mutation burden is associated with the efficacy of immune checkpoint blockade treatment [6]. However, mutation-derived neoantigens are often specific to an individual, and the frequencies of non-synonymous mutations vary among cancer types [7]. Furthermore, consistent with the cancer immunoediting theory [8], the immunopeptidomes of neoantigens are subject to active immune responses: strong neoantigens can be “edited out” [9]. Indeed, tumors that develop in hosts with intact immune responses are usually less immunogenic than those that arise in an immunodeficient microenvironment [10]. In other words, as the functional T cell response controls tumor growth, it also promotes the selection of tumor cells expressing less immunogenic tumor antigens, thereby promoting escape from immune responses [9]. However, the remaining unedited neoantigens may be sufficient to mediate tumor regression with the application of appropriate immunotherapies.

Interestingly, by tracking TCR clones during the development of tumors, a recent study demonstrated that tumor-infiltrating CD8 T cells exhibit dynamic clonotype transitions [11]. Of note, this clonal replacement plays a key role in the success of anti-PD-1 blockade treatment. These data suggest that tumor-specific CD8 T cells respond in several “waves,” with different clonotypes that recognize and adapt to the tumor in distinct rounds of cancer immunoediting [2, 3]. More importantly, these data also indicate that anti-tumor responses require collaboration and coordination among various immune cell subsets. In this review, we will focus on how tumor-infiltrating T cells may undergo desensitization of their TCR signaling machinery, how T cell cross-talk with dendritic cells (DCs) launches T cell immune responses against malignancies, and how the tumor microenvironment (TME) can change the dynamics of cell–cell interactions.

## Intrinsic defects in TCR signaling in tumor-infiltrating T cells

For successful T cell activation, a T cell must first recognize an antigen presented by an antigen-presenting cell (APC). The TCR:pMHC interaction then triggers intracellular signaling networks, eventually resulting in T cell commitment to activation. Therefore, the presentation of tumor neoantigens on APCs, which drives the expansion of tumor-reactive T cells, is essential to initiation of the T cell response. Full T cell activation requires synchronization of TCR signals (signal 1), costimulatory or coinhibitory receptor signals (signal 2), and/or cytokine signals (signal 3). TCR recognition of a pMHC signal is a dominant factor in commitment to activation, and signals 2 and 3 may synergize with or antagonize signal 1, to either augment or block T cell effector function and fate decision [12–16]. Fully activated T cells will be able to expand and differentiate into cytotoxic and helper T cells, and then migrate into peripheral tissues to seek their target cells. In the context of tumors, however, infiltrating T cells are often found to be dysfunctional [17]. As posited in the cancer immunoediting theory, while lymphocytes prevent and control tumor growth, continuous immune responses within developing tumors may also favor the development of immunologic anergy, tolerance, or even exhaustion. In recent years, considerable research efforts have been directed toward understanding the molecular mechanisms of T cell dysfunction in tumors.

The initiation of TCR signaling is mainly controlled by two key kinases, the Src family kinase LCK and ζ chain-associated protein kinase 70 (ZAP-70) [16, 18]. Recognition of antigen pMHC triggers LCK to phosphorylate CD3 or the ζ chains on the tyrosine residues of their immunoreceptor tyrosine-based activation motifs (ITAMs). Doubly phosphorylated ITAM motifs recruit the kinase ZAP-70 to the engaged TCR complexes. ZAP-70 is subsequently phosphorylated and activated by LCK. Activated ZAP-70 phosphorylates the adaptor proteins linker for activation of T cells (LAT) and lymphocyte cytosolic protein 2 (SLP76). Both LAT and SLP76 function as signal amplifiers. Upon phosphorylation, LAT and SLP76 initiate diverse downstream cellular signaling events that lead to numerous cellular functions, including cell migration, calcium mobilization, cytokine production, effector functions, and cell proliferation. In addition, LAT-mediated calcium–NFAT signaling can promote exhaustion and anergy.

Interestingly, T cells are hardwired with many inhibitory signal networks to ensure that they can be activated only by a bona fide signal. These networks allow T cells to return to their basal state once the abnormal (or infected) cells are eliminated and help avoid immunopathology. In other words, the activating signal mechanisms endow T cells with the sensitivity to quickly respond to a systemic disturbance, and the inhibitory signal mechanisms dampen T cell effector responses to maintain the balance of the immune system and prevent immunopathology. In tumor-infiltrating T cells, many signaling pathways are disturbed or impaired; for example, proximal TCR signaling molecules are downregulated or even lost, and inhibitory receptors are upregulated. Thus, the balance of activating and inhibitory signal networks is disturbed in tumor-infiltrating T cells, which prevents robust anti-tumor T cell responses. Recent studies employed genome-wide CRISPR screens to provide insights into the key molecules with activating and inhibitory signal domains that regulate T cell proliferation and effector functions [19, 20]. The study ranked the top eight key signaling mediators that critically regulate T cell proliferation [19]. The essential activating signal domains in TCR proximal signaling molecules were identified in SLP76, LAT, LCK, CD3, and ζ chain. The key inhibitory signal domains appeared in signaling molecules such as the E3 ubiquitin ligase Cblb, suppressor of cytokine signaling 1 (SOCS1), diacylglycerol kinase α (DGKA) and ζ (DGKZ), tyrosine-protein phosphatase non-receptor type 2 (PTPN2), and JUN [19]. Importantly, blocking these inhibitory signals boosted anti-tumor killing capacity in vitro [19]. Many of these candidate genes are also found in the anergy or exhaustion gene signatures identified in tumor-infiltrating T cells [20–24]. The data thus provide a rich resource for navigating the molecular basis of tumor-infiltrating T cell dysfunction and a framework for identifying potential targets to reinvigorate tumor-infiltrating T cell anti-tumor responses [19, 20].

T cell activation often leads to the downregulation/internalization of TCR complexes, as well as the upregulation of an additional set of inhibitory receptors, signaling molecules, and transcription factors. These sets of inhibitory signaling proteins—including cytotoxic T lymphocyte attenuator (CTLA-4), B and T lymphocyte attenuator (BTLA), and programmed cell death protein 1 (PD-1)—are employed to fine-tune T cell effector responses and participate in “negative feedback loops” [25]. The upregulation of CTLA-4 and PD-1 are of substantial interest [26, 27], due to the therapeutic success of anti-CTLA-4 and anti-PD-1 blockade [28]. The expression of CTLA-4 is immediately upregulated after T cell activation [29], enabling it to regulate the T cell activation phase. CTLA-4 competes with higher affinity than CD28 for the shared ligands CD80 and CD86 [30–32]. Moreover, upon binding to CD80 and CD86, CTLA-4 is able to mediate trans-endocytosis to deplete these two ligands on the APC surface, thereby further hampering CD28 costimulatory signaling [33, 34]. PD-1 expression is induced later than CTLA-4 in activated T cell subsets, facilitating regulation of the effector phase of T cell responses. PD-1 has two ligands, PD-L1 and PD-L2. Upon binding to PD-L1, PD-1 selectively recruits the protein tyrosine phosphatase Src-homology-2 domain containing phosphatase 2 (SHP2) to profoundly blunt T cell proliferation and cytokine production [35]. Interestingly, CD80 and PD-L1 on APCs can also interact with each other in cis as CD80–PD-L1 heterodimers, which restricts CTLA-4 and PD-1 inhibitory signaling [36, 37]. Taken together, these findings suggest that anti-CTLA-4 immune blockade enhances CD28-mediated costimulatory signals in T cells to promote T cell priming, whereas anti-PD-1 and anti-PD-L1 immune blockade reduce PD-1–mediated inhibitory signals to promote T cell effector function.

Not only do CTLA-4 and PD-1 employ distinct molecular machineries, but anti-CTLA-4 and anti-PD-1 blockade operate via distinct cellular mechanisms. Anti-CTLA-4 checkpoint blockade predominantly promotes Th1-like CD4 T cell responses, whereas anti-PD-1 blockade preferentially enhances the expansion of phenotypically exhausted CD8 T cells [28, 38]. Consistent with their blockade through non-overlapping mechanisms, anti-CTLA-4 and anti-PD1 therapies work synergistically in clinical studies. However, despite the success of immune blockade therapies in various cancers, only a small subset of patients benefit from these strategies, necessitating continuous research efforts to identify additional targets––including V-domain Ig-containing suppressor of T cell activation (VISTA) [39], BTLA [40], and lymphocyte activation gene 3 (LAG3)––and develop new immune-blockade therapies. So far, successful immune blockade therapies have targeted inhibitory modules that regulate T cell sensitivity to tumor-specific neoantigens, providing a therapeutic opportunity to promote potent anti-tumor T cell responses. An effective T cell immune response requires mature APCs to process and present cognate antigens to deliver a bona fide activating TCR signal to initiate T cell responses. The costimulatory and coinhibitory molecules displayed on APCs also modulate the quality of TCR signals. Indeed, recent studies also suggest that DCs may play essential roles in instructing anti-tumor immune responses [41, 42].

## Factors within the TME that modulate tumor-infiltrating T cell function

In addition to T cell interactions with other immune cells that can modulate TCR signaling and resulting T cell function, molecules within and/or features of the TME may also affect T cell activation and responses. These signaling factors include cytokines that are produced by tumor-infiltrating immune cells, which can be either pro-inflammatory (i.e., IFN-γ) or immunosuppressive (i.e., TGF-β) [43]. Recent studies have highlighted that IFN-γ production by intratumoral T cells plays a key role in mounting anti-tumor immune responses, and checkpoint blockade can also augment anti-tumor responses and increase T cell effector gene signatures through IFN-γ–driven remodeling of T cells [24].

Furthermore, pleiotropic cytokines such as IL-2 and IL-10, despite being both immune stimulatory and suppressive, are also important, and have revealed promise as therapeutic targets that can be combined with checkpoint blockade therapies. IL-2 is a T cell growth factor and can enhance T cell survival and function [44, 45]. However, it can also stimulate regulatory T cells, enhance immune suppression, and potentially result in severe toxicities. Recently, engineering T cells with orthogonal IL-2 cytokine–receptor complexes has proven to be an efficient way to selectively expand T cell populations of interest with minimal toxicities, and the resulting expanded T cells retain anti-tumor effector function and effectively control the growth of syngeneic B16-F10 tumors in mice [45]. Another pleiotropic cytokine, IL-10, has also been shown to reinvigorate terminally exhausted T cells. Although IL-10 is usually considered an immunosuppressive cytokine and higher IL-10 expression correlates with tumor progression, a recent study provided compelling evidence that an engineered interleukin-10–Fc fusion protein may act through metabolic rewiring to enhance the effector functions of terminally exhausted CD8 T cells [46].

Metabolites that are enriched in the TME can also serve as key players to influence T cell responses, including indoleamine 2,3-dioxygenase (IDO) and adenosine [47]. Adenosine is of particular interest. As an immunosuppressive factor, adenosine is generated by the ectoenzymes CD73, CD39, and CD38, which are often highly expressed in anergic, exhausted, or regulatory T cell subsets that limit T cell immune responses [47]. In healthy tissues, the concentration of extracellular adenosine is low. Cell death, hypoxia, and persistent inflammation within the TME can all lead to the efflux of ATP and result in increased concentrations of extracellular adenosine. Extracellular adenosine starts as a nucleotide that undergoes stepwise dephosphorylation from ATP to ADP to AMP, in processes mediated by ectonucleotidases (i.e., CD39), before finally being converted to adenosine (e.g., by CD73). Adenosine-mediated T cell suppression primarily occurs through binding to the adenosine receptor A_2A_R, resulting in the activation of adenylate cyclase, and, most importantly, the accumulation of intracellular cyclic AMP [48]. Accumulated intracellular adenosine may mediate potent immunosuppressive function in T cells. By sustaining protein kinase A (PKA) activation, elevated intracellular cyclic AMP dampens TCR proximal signaling and CD28-mediated costimulatory signals, and even enhances other negative regulatory domains, including Csk and SHP-1 [49]. Indeed, targeting adenosine/adenosine receptors (i.e., A_2A_R) can enhance anti-tumor T cell responses and enhances chimeric antigen receptor (CAR) T cell efficacy [50, 51]. Along the same line, antibodies that target ectoenzymes, such as CD73, have also recently showed promising clinical results [52].

Last, the acidity of the TME may also profoundly suppress T cell effector functions. While the low pH may not hinder the initial response between T cells and DCs, the acidic microenvironment substantially inhibits T cell proliferation, decreases IL-2 and IFN-γ secretion, and reduces T cell glycolysis. Buffering the acidity within the TME has also been explored to improve T cell anti-cancer therapies. Interestingly, a pH-sensitive signaling pathway is important in regulating T cell activation, and has recently been suggested to be a naturally occurring mechanism to balance between bona fide activation and avoiding unwanted immune responses. How the pH-sensitive signaling pathway in T cells may contribute to anti-tumor immune responses requires further investigation.

## MHC class II neoantigens are required for successful CD8 T cell anti-tumor responses through the recruitment of CD4 help

In animal models, a successful anti-cancer immune response does not simply rely on MHC-I–restricted neoantigens to drive tumor antigen-specific CD8 T cell responses; the presentation of neoantigens on MHC class II (MHC-II) molecules to CD4 T cells within tumors also plays an essential role [4, 53, 54]. Interestingly, similar results have been observed in human tumors [55], in which neoantigens were presented by human MHC-II (leukocyte antigen class II molecules), particularly on APCs [55]. Unlike MHC-I molecules, which are expressed and can be upregulated on all nucleated cells (including tumors), MHC-II molecules are primarily expressed on APCs. MHC-II–restricted neoantigens recruit tumor-specific CD4 T cells to tumor sites, subsequently promoting optimal priming and generation of CD8 cytotoxic T lymphocytes to eliminate tumor cells [4]. Moreover, CD4 T cells secrete IFN-γ, driving the remodeling of CD4 and CD8 T cells, and enhancing their anti-cancer effector functions [24]. These data, therefore, suggest that CD4 and CD8 T cells play non-redundant roles during anti-tumor responses. More importantly, these studies support the concept that both MHC-I– and MHC-II–restricted neoantigens need to be processed and presented by APCs, indicating that APCs shape anti-tumor immunity by coordinating the collaboration of CD4 and CD8 T cells.

Given the critical role of DCs in simultaneously tailoring CD4 and CD8 T cell responses during different steps in the anti-tumor immune cycle, the question becomes, how does the TME affect DC physiology and function? In the following sections, we will discuss how the maturation, migration, and function of DCs are influenced by tumors.

## DCs play a central role in coordinating CD4 and CD8 T cell anti-tumor responses

**Fig. 1:**
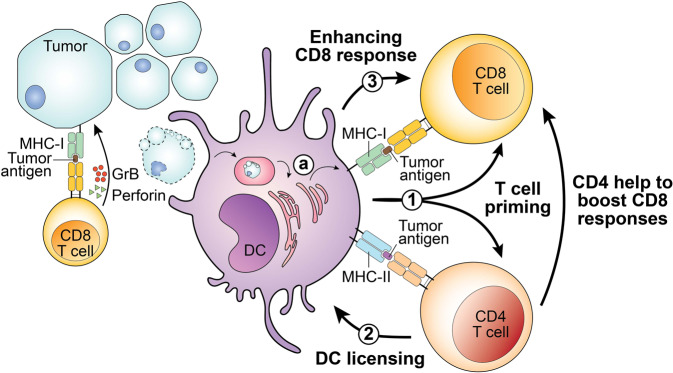
Conventional dendritic cells coordinate CD4 and CD8 T cell anti-tumor immune responses. Tumor cells may process and present tumor-specific or -associated antigens on MHC class I molecules through the MHC-I antigen processing pathway. CD8 T cells recognize tumor antigens presented by tumor cells and may secrete granzyme B (GrB) and perforin to mediate cytotoxicity toward tumor cells. In addition, dendritic cells (DCs), particularly the conventional DC1 (cDC1) subset, play several roles in helping CD4 and CD8 T cells to target tumors. **1** DCs may process and present or cross-present, shown in (**a**) tumor-derived MHC-I– and MHC-II–restricted tumor antigens to CD8 and CD4 T cells, respectively. T cells that can recognize tumor-specific neoantigens bind to the neoantigen peptide:MHC complex, and become activated, in a process termed T cell priming. **2** Activated CD4 T cells can “license” cDC1 cells through CD40:CD40L signals. **3** Subsequently, cDC1 cells can further enhance CD8 T cell activation. In addition to indirectly helping to boost CD8 responses through cDC1-mediated licensing, CD4 T cells can also provide direct help to augment CD8 T cell responses.

DCs can be categorized based on their morphology, development, function, and key transcription factors and phenotypic markers. Conventional type I DCs (cDC1s) are responsible for initiating CD8 T cell responses [56], whereas type II DCs (cDC2s) mainly initiate CD4 responses [57, 58]; CD4 T cells also help “license” cDC1s through CD40 signals. Consequently, CD4 T cells increase the antigen presentation and costimulatory functionality of DCs to boost and maximize CD8 T cell function [59]. A recent finding provides further direct evidence that cDC1s relay CD4-mediated “help signals” to boost CD8 T cell effector functions against tumors [41] (Fig. 1). Using a highly immunogenic fibrosarcoma model, the study showed that cDC1s simultaneously prime and initiate CD8 and CD4 T cell responses [41]. Subsequently, activated CD4 T cells provide direct help to CD8 T cells through secreted molecules, and license cDC1s to further assist CD8 responses in a cDC1-dependent manner [41]. Therefore, cDC1s not only induce T cell responses, but orchestrate cross-talk between CD4 and CD8 T cells. Preventing or restricting MHC-II molecule expression to only cDC1s further demonstrated that CD4 T cells need to directly engage cDC1s for optimal priming [41]. This functional interaction between CD4 T cells and cDC1s requires TCR:pMHC-II recognition in the initial phase, and CD40:CD40L signaling for cDC1 licensing.

In addition to cDC1s, which orchestrate the cross-talk between CD4 and CD8 T cells to achieve a potent T cell response, plasmacytoid dendritic cells (pDCs) are emerging as important regulators of anti-tumor responses. pDCs produce large amounts of type I IFN, which positively acts on cDC1s by inducing their maturation [60]; promoting expression of CCR7 and, therefore, migration to the lymph node; and increasing their cross-priming ability [61]. In a model of acute infection, CCR5-expressing pDCs are recruited to the cDC–CD8 T cell interaction site via the CCL3 selectively produced by activated CD8 T cells, and the type I IFN produced locally by pDCs enhances cDC1 licensing ability, resulting in optimal T cell responses [62]. Interestingly, the adoptive transfer of tumor antigen-loaded pDCs induces a potent anti-tumor T cell response in melanoma patients [63]. This could be due to the cross-talk with cDC1 and CD8 T cells described in the infection model or to a role as APCs. pDCs, in fact, have been shown to directly prime melanoma-specific CD8 T cells [64], but also to cross-prime CD8 T cells by transferring antigen to cDC1s [65].

However, the role of pDCs in the TME remains contradictory. Tumor-infiltrating pDCs are immature and hypofunctional, and they have been correlated with a negative clinical outcome. Tumor-derived factors, such as TGF-β and prostaglandin E2 (PGE2), alter pDC functionality by inhibiting their ability to produce type I IFN [66], and tumor cells expressing Wnt5a inhibit pDC activation and IFN-α secretion [67]. Moreover, intratumoral pDCs express OX40L, and its interaction with T helper cells induces IL-5/IL-13 production, which has been shown to foster tumor progression [68]. In addition, IDO and ICOS-L expression on tumor-infiltrating pDCs leads to regulatory T cell-mediated immunosuppression of anti-tumor T-cell immunity [69, 70].

## Defective DC migratory properties compromise T cell anti-tumor responses

The draining lymph node is the major site of the cell–cell interactions required to initiate a T cell response. Interestingly, an increasing number of studies has demonstrated that ectopic lymphoid formations in tumors, termed tertiary lymphoid structures, are associated with clinical outcome, including patient survival and responses to immune checkpoint blockade, in various cancers [71]. Although they can vary vastly in cellular composition and structure, tertiary lymphoid structures in cancers facilitate the clustering of multiple cell subsets, including T cells, B cells, and DCs, into germinal center-like structures that promote and sustain anti-tumor immune responses [72]. The observation of tertiary lymphoid structures in the tumor setting highlights that an effective anti-tumor response requires a productive collaboration between the adaptive and innate immune systems and unveils the importance of DCs in orchestrating effective T cell anti-tumor responses [73]. However, further studies are warranted to generate a detailed picture of how these tertiary lymphoid structures may support T cell–cDC1 interaction within tumors, and whether the temporal resolution and spatial location of these cell–cell interactions are similar to those observed in lymph nodes during infection.

DC migration back to lymph nodes after antigen collection in tumors is the fundamental step that launches T cell anti-tumor immunity. The migratory pathways in DCs are regulated by interactions between chemokine receptors on DCs and chemokines present in the tissue microenvironment. Tumor mutations and suppressive molecules are responsible for DC exclusion from tumors. Typically, cDC1s migrate into tumors via the interaction of the chemokine receptor C-C chemokine receptor type 5 (CCR5) with its ligands C-C motif chemokine ligand 4 and 5 (CCL4 and CCL5), and X-C motif chemokine receptor 1 (XCR1) with its ligand X-C motif chemokine ligand 1 (XCL1). In melanoma, aberrantly active β-catenin signaling in melanoma cells suppresses CCL4 expression to abolish cDC1 infiltration [74]. Moreover, the downregulation of uncoupler protein 2 (UCP2) expression in melanoma cells has been shown to abrogate chemokine-induced cDC1 tumor infiltration [75].

**Fig. 2:**
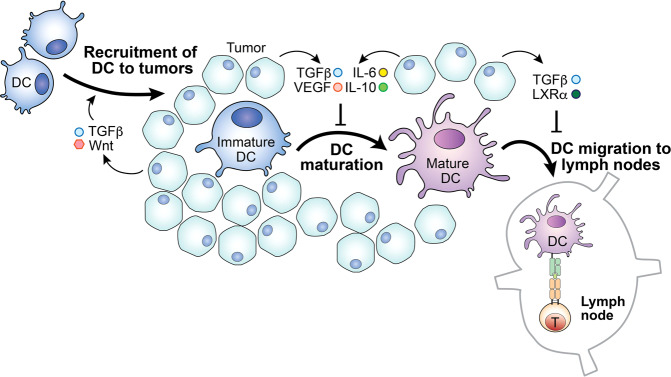
The tumor microenvironment alters dendritic cell function. The tumor microenvironment (TME) may inhibit dendritic cell (DC) functions, abrogating DCs’ ability to promote T cell responses. Tumor cells often upregulate the expression of CD47, which may inhibit DC presentation of antigens to T cells. In addition, the accumulated lipid and metabolites within the TME exhibit similar negative effects on DC antigen presentation. Moreover, the elevated concentrations of TGF-β and Wnt, as well as LXRα, promote DC migration to the tumor but block migration to the lymph nodes. Furthermore, the TME is rich in cytokines IL-6, IL-10, and TGF-β, as well as tumor-derived VEGF. These molecules inhibit DC maturation, altering their ability to initiate a T cell response.

The accumulation of the immunosuppressive molecule PGE2 in the TME, as a result of an increased abundance of immunosuppressive cells and production by cancer cells, has also been shown to impede cDC1 migration into tumors [76]. After collecting tumor antigens, cDC1s migrate to the draining lymph node, where they can activate T cells, in a process mediated by CCR7 expression. Interestingly, TGF-β, which is secreted by tumor cells and tumor-infiltrating regulatory T cells [77–79], enhances the expression of chemokine receptors CCR1, CCR3, CCR5, CCR6, and CXCR4 on DCs, thereby driving DC migration to the TME. On the other hand, TGF-β signaling also decreases the expression of CCR7 on DCs. As a result, robust TGF-β signaling traps DCs within the tumor and impairs T cell priming in the lymph node [80] (Fig. 2). Of note, the accumulation of cDC1s in tumors also plays a critical role in supporting T cell tumor infiltration via their production of CXCL9 and CXCL10. Thus, the lack of cDC1s in tumors leads to insufficient priming, expansion, and tumor infiltration of tumor-specific T cells, which cumulatively compromise patient responsiveness to PD-1 blockade treatment. However, forced accumulation of immature cDC1s in tumors can also impede T cell immune responses against malignancy. Therefore, understanding how these sequential events are coordinated, in order to support the migration of cDC1s in and out of tumors, could be a critical foundation for developing strategies to fire up cold tumors and boost responsiveness to PD-1 blockade treatment.

## Microenvironmental hurdles hamper DC maturation in tumors

Proper activation is critical for DC maturation, antigen loading and presentation, and homing ability to the draining lymph nodes. DC activation requires the engagement of various pattern recognition receptors, including sensors of cytosolic DNA. DCs can take up DNA derived from dying cancer cells, which then activates the cyclic GMP-AMP synthase-stimulator of interferon genes (cGAS-STING) pathway and results in type I IFN production. The production of type I IFN, in turn, supports DC cross-priming of tumor-specific T cells, which is required for potent CD8 T cell anti-tumor immunity [61, 81]. Interestingly, the expression of CD47 on cancer cells not only provides “do not eat me” signals, but also has been shown to interfere with antigen cross-presentation by cDCs by interrupting cGAS-STING activation [82]. Mechanistically, CD47 expressed on tumor cells interacts with signal-regulatory protein-α (SIRPα) on cDCs to facilitate the recruitment of the tyrosine phosphatase SHP-1 on the phagosomal membrane. As a result of this signaling complex formation, SHP-1 inactivates the NADPH oxidase, NOX2, thereby expediting phagosome acidification and accelerating cancer cell mitochondrial DNA (mtDNA) degradation. In this context, engulfment of cancer cells is unable to activate the cGAS-STING-interferon regulatory factor 3 (IRF3) axis or induce type I IFN production to augment the ability of DC1s to cross-present antigens. Upon CD47 blockade treatment, which mitigates the NOX2-mediated reduction of tumor mtDNA degradation, DCs can restore cross-presentation of tumor antigens [83, 84].

In addition to the “do not eat me” signal, aberrant lipid accumulation in tumor-infiltrating DCs, caused by upregulation of the scavenger receptor Msr1 (CD204) and engagement of endoplasmic reticulum (ER) stress responses, robustly impedes the ability of DCs to prime T cells [85, 86]. Mechanistically, excessive lipid loading in DCs impairs external antigen processing and the cross-presentation of peptide on MHC-I complexes [87]. In particular, truncated and oxidized fatty acids bind to the 70-kDa heat shock protein (HSP70) and force its interaction with lipid bodies instead of lysosomes, which blocks cross-presentation [88]. In addition to high lipid content, tumor-infiltrating DCs have high reactive oxygen species (ROS) levels, which cause intracellular lipid oxidation and byproduct generation, resulting in ER stress. In the setting of ER stress, the stress sensor inositol-requiring enzyme 1 (IRE-1) activates X-box binding protein 1 (XBP-1). The activation of ER stress responses leads to lower surface expression of pMHC-I and decreased capacity to induce T cell proliferation [86]. Other metabolites in tumors, including lactic acid, can also dampen antigen presentation capability and block IL-12 and IFN-α production in DCs [89]. Mechanistically, the exposure of DCs to lactate causes a dramatic decrease in endosomal pH and accelerates proteolytic cleavage, which interfere with antigen presentation [90].

The activation and maturation of DCs can also be suppressed by efferocytosis, a process that restrains pro-inflammatory responses in phagocytes that have engulfed apoptotic cells [91]. During efferocytosis, DCs upregulate the expression of receptor tyrosine kinases, including MER proto-oncogene, tyrosine kinase (MerTK), AXL receptor tyrosine kinase (Axl), and tyrosine-protein kinase receptor Tyro3, which in turn stimulates immunosuppressive polarization. Moreover, the engulfment of apoptotic cancer cells can stimulate peroxisome proliferator activated receptor γ (PPARγ) and the liver X receptor α (LXRα), driving the polarization of immunosuppressive APCs by reprogramming metabolic processes. Hence, the extrinsic and intrinsic metabolic signals in tumor-infiltrating DCs can impede initiation of T cell anti-tumor immunity.

Cytokines have also been shown to alter the DC phenotype in tumors. Tumor-derived vascular endothelial growth factor (VEGF) can inhibit FMS-like tyrosine kinase 3 ligand (FLT3L) activity [92]. Since FLT3L is a key factor for DC development, proliferation, and maturation [92], VEGF-mediated inhibition of FLT3L negatively affects cDC differentiation in vivo. Importantly, neutralizing VEGF produced by cancer cells can restore the functional maturation of DCs derived from human CD34^+^ cord blood cells in vitro [93], further supporting the importance of VEGF-mediated signals in tumor-infiltrating DC maturation. On the molecular level, VEGF may prevent DCs from acquiring a mature phenotype via inhibition of the transcription of NF-κB, which is needed for DC maturation and T cell stimulatory ability [94].

Tumors are rich in IL-6 and IL-10, and the tumor-derived TLR-2 ligand versican can induce the overexpression of IL-6 and IL-10 receptors on DCs [95, 96]. In multiple myeloma patients, the elevated IL-6 levels within the TME not only alter the development, number, phenotype, and function of DCs, but also direct the differentiation of monocytes into macrophages rather than DCs [97, 98]. This reduction in DC maturation can be explained by the IL-6/IL-10–STAT3 signaling axis. IL-6 and IL-10 signaling promote STAT3 activation, which fosters IL-10 production at the expense of IL-12 production in DCs. Meanwhile, the expression levels of MHC-II, CD40, and CD86 are also downregulated, rendering DCs more immunosuppressive [99]. TGF-β has been found to regulate versican synthesis in fibrosarcoma, osteosarcoma, and glioma cells [100–102]. Thus, in addition to impairing DC recruitment, suppressing DC maturation, and inhibiting the production of TNF-α, IL-12, and IFN in DCs, TGF-β also promotes the development of immature and tolerogenic DCs, contributing to impaired T cell anti-tumor responses by inducing IL-10 and further TGF-β production [103, 104]. Furthermore, TGF-β induces the upregulation of IDO, an enzyme associated with immunosuppressive functions, which also contributes to the induction of tolerogenic DCs [105, 106]. Taken together, these findings suggest that interventions targeting those microenvironmental hurdles may represent a new arsenal for unleashing T cell anti-tumor immunity by reprogramming the immune status of the TME.

## Conclusion and future directions

Canonical TCR signaling is initiated by the phosphorylation of tyrosine residues on a few key proteins, most notably the intracellular tails of the T cell receptor and ζ chain, and LAT. Phosphorylation of these proteins creates docking sites for the assembly of multi-component signaling complexes that coordinate Ca^2+^ mobilization, cytoskeletal rearrangement, MAP kinase signaling, and other processes.

Signaling dysfunction compromises the ability of tumor-infiltrating T cells to efficiently elicit a T cell response. Tumor-infiltrating T cells often have impaired ability to evoke activation signals, and their inhibitory signaling domains are upregulated. Many promising anti-cancer immunotherapies have been based on the premise that intervening in the TCR signaling network may reinvigorate these dysfunctional T cells. For example, tumor-infiltrating T cells express high levels of the coinhibitory molecules PD-1 and CTLA-4, thereby enabling immune checkpoint blockade therapies to prevent the ligand-binding–induced inhibitory signals. Recently, an elegant study engineered a bi-specific molecule to recruit the phosphatase CD45 to be in close proximity to PD-1 [107]. This special recruitment of CD45 can force the intracellular phosphatase domain of CD45 to dephosphorylate the tyrosine of PD-1’s signaling motif, suppressing PD-1–mediated inhibitory signals [107]. Overall, modulation of the signaling properties of these coinhibitory receptors has shown therapeutic promise.

Another prime example of leveraging our knowledge in TCR signaling to treat cancer is the development of CAR T cells [108]. Early CAR designs were based on the premise that incorporation of the intracellular region of the ζ chain into a CAR is sufficient to recruit ZAP-70, evoke TCR signaling, and elicit a T cell response. CARs are engineered proteins that consist of an extracellular antigen-binding domain (often a single-chain antibody variable fragment, scFv) fused to TCR signaling components. Typically, the signaling motifs of CARs contain two parts: a CD3 or ζ chain segment to engage TCR signals (signal 1) and a CD28 or 4-1BB segment to engage costimulatory signals (signal 2) [109]. Despite many successes using this therapeutic modality, challenges still remain in the design of highly efficacious CAR T cells. Studies have shown that even these next-generation CARs induce signaling that is not identical to, and often not as robust as, canonical TCR signaling. For instance, CAR T cell therapy can effectively treat certain liquid tumors; however, its application to solid cancers remains limited partly due to low CAR T cell activity and maintenance in vivo in this context. These artificial receptors appear to be much less efficient at detecting foreign antigens than endogenous TCRs, and only partially engage canonical TCR pathways for T cell activation [110–114]. Moreover, scFv-based CAR affinities are logs-fold higher than those of endogenous TCRs, and constant antigen stimulation and tonic signaling in vivo also contribute to the defects in CAR T cell activity and maintenance [115, 116]. Indeed, studies have shown that by manipulating how CARs adapt to antigen stimulation [117–119] or are maintained by tonic signaling [116], we can further engineer a more potent CAR T cell to improve therapies [120].
